# ALLMAPS: robust scaffold ordering based on multiple maps

**DOI:** 10.1186/s13059-014-0573-1

**Published:** 2015-01-13

**Authors:** Haibao Tang, Xingtan Zhang, Chenyong Miao, Jisen Zhang, Ray Ming, James C Schnable, Patrick S Schnable, Eric Lyons, Jianguo Lu

**Affiliations:** Center for Genomics and Biotechnology, Fujian Agriculture and Forestry University, Fuzhou, 350002 Fujian Province China; School of Plant Sciences, iPlant Collaborative, University of Arizona, Tucson, AZ 85721 USA; Data2Bio LLC, 2079 Roy J. Carver Co-Lab, Ames, Iowa 50011 USA; J. Craig Venter Institute, 9704 Medical Center Dr, Rockville, MD 20850 USA; Department of Agronomy and Horticulture, University of Nebraska, Lincoln, NE 68588 USA; Department of Agronomy, Iowa State University, Ames, IA 50011 USA; Heilongjiang River Fisheries Research Institute, Harbin, 150070 China

## Abstract

The ordering and orientation of genomic scaffolds to reconstruct chromosomes is an essential step during *de novo* genome assembly. Because this process utilizes various mapping techniques that each provides an independent line of evidence, a combination of multiple maps can improve the accuracy of the resulting chromosomal assemblies. We present ALLMAPS, a method capable of computing a scaffold ordering that maximizes colinearity across a collection of maps. ALLMAPS is robust against common mapping errors, and generates sequences that are maximally concordant with the input maps. ALLMAPS is a useful tool in building high-quality genome assemblies. ALLMAPS is available at: https://github.com/tanghaibao/jcvi/wiki/ALLMAPS.

## Background

A hierarchical approach is typically adopted for the assembly of large eukaryotic genomes - starting with identifying overlapping reads to build contigs, then adding paired reads to build scaffolds, and finally ordering and orientating scaffolds together to assemble chromosomes using various sources of long distance information [1,2]. During this hierarchical process, larger and larger sequence chunks are assembled and finally ‘anchored’ onto chromosomal-sized pieces. The reconstructed chromosomal sequences are often referred to as ‘pseudo-molecules’ or ‘pseudo-chromosomes’ [3,4]. The prefix ‘pseudo’ implies that the assemblies may still contain uncertainties, and only represent a single specific hypothesis that needs to be evaluated in view of all available evidence [5].

One of the major steps in producing high quality genome assemblies is to use a variety of mapping information, including genetic maps, physical maps, cytological maps, optical maps, or synteny with related taxa to reconstruct the most likely chromosomal assemblies [4,6-10]. Many specialized tools have been developed over the years to assist with the various steps of the hierarchical genome assembly pipeline, including contigging [11], scaffolding [1,2], optical map alignment [9,12], and synteny-guided assembly [4,13]. In comparison, tools for anchoring of scaffolds based on more than one genetic map remain under-developed. Our method ALLMAPS, fills this algorithmic gap by optimizing agreement among multiple maps to order and orient scaffolds. While initially designed to use genetic maps to guide the chromosomal anchoring process and produce a close-to-optimal scaffold configuration, ALLMAPS can utilize a variety of techniques for generating physical and comparative maps of chromosomes.

A key feature of ALLMAPS is its built-in ability to handle multiple maps in a unified framework. Organisms that have been subjected to a moderate amount of research often have several genetic maps available, likely constructed by different labs at different points in time. These genetic maps are often derived from different mapping populations using genetically diverse parental lines, making consensus mapping across multiple maps difficult or sometimes infeasible [14]. Maps constructed from F1 population derived from two heterozygous individuals typically produce two genetic maps, one for each gender, based on a backcross model [15,16]. Maps constructed using different software, such as R/QTL [17], MSTMAP [18], and JOINMAP [19], often differ in their final ordering of genetic markers. Recent Genotyping-by-Sequencing (GBS) technology has been used to generate high-density genetic maps through the use of multiplexing and high throughput sequencing [20]. There is a pressing need to incorporate all available mapping information during the construction of chromosomal assemblies for organisms of interest, irrespective of differences due to the population, employed markers, or mapping software.

There are several benefits of using multiple genetic maps for scaffold anchoring. Genetic maps can vary in terms of recombination frequency, segregation distortion, presence-absence variation (PAVs), and chromosome regions with few mapped polymorphic sites. If such differences among maps are complementary, it may be possible to anchor scaffolds on one map that cannot be anchored on another. In addition, combining evidence from multiple maps to create a ‘meta-order’ can address potential weaknesses intrinsic in any single map. The assimilation of different maps means that many legacy maps (even those created by different labs) can be utilized to support the final scaffold order of a common reference genome assembly. In summary, multiple independent maps can provide more accurate scaffold orientation and ordering information, allowing a greater portion of the scaffolds to be anchored than would be possible with any single map alone.

The use of a single map to guide scaffold ordering is a computationally trivial problem. The scaffolds could simply be sorted based on the average map locations of the markers on each scaffold and there would be little ambiguity in the reconstructed order. In contrast, the goal of ALLMAPS is to combine information from multiple genetic maps to compute a common scaffold order. This is a more difficult challenge. Multiple maps may suggest incompatible scaffold orders and orientations, or ‘conflicts’. Computationally, each map implies a ‘partial’ scaffold ordering, which can be modeled as a directed acyclic graph (DAG), with edges representing the relative order between scaffolds. In the absence of conflict, the order of physical scaffolds can be solved by merging multiple DAGs followed by a topological sort. When conflicts exist between various maps, the resulting merged graph will contain conflicts, which must be resolved before direct sorting.

To build a consensus order, clear and objective rules for conflict resolution are necessary. Conflict resolution among input maps is conceptually similar to the problem of building consensus maps [14], but with important differences. In the case of building a consensus genetic map, a common set of markers must be present across most of the genetic maps in consideration. In general, we cannot expect to build a consensus map first and then use the consensus map to guide the scaffold anchoring. Even in the case when a consensus map is possible, too much information may be lost during the process of building consensus map, which itself is a difficult problem to solve [14]. Further, in some applications especially in the case of maps generated via GBS [20], pairs of maps may share few or no markers but each genetic map can contain markers that are shared with the physical scaffolds.

Many studies making use of multiple maps employ *ad hoc* rules to resolve conflicts in the scaffold ordering among maps [15]. While some of these rules may be effective in a particular study, they are inherently subjective and can become difficult to extrapolate to different genome datasets and replicate in future studies. In other cases, scaffold ordering may require arbitrary human decisions in regions of conflicting evidence [3,21]. As an increasing number of genome projects have access to multiple mapping data resources [3,8,10,21,22], a method that could accurately combine several lines of evidence to build high quality chromosomal assemblies has become essential.

Here, we implement a novel method, ALLMAPS, to address the current lack of computational tools for performing objective scaffold ordering based on colinearity of multiple maps. Colinearity, defined as the arrangement of one sequence in the same linear order as another sequence, is one of the most important criteria in evaluating map concordance [3,8,16] and evolutionary relatedness [4,23].

We highlight several salient features of ALLMAPS. First, we have formulated a clear, computable objective which is to maximize the sum of colinearity to multiple input maps, leading to better reproducibility in the anchoring process. Second, we allow variable weights in input maps, leading to better control in conflict resolution between different maps. Finally, we show that ALLMAPS can naturally be extended to incorporate other mapping evidence, including optical map and cross-species synteny, requiring minimal effort of data transformation. ALLMAPS is an elegant tool that promises to expedite genome assembly and facilitate the integration of various mapping evidence during the final stage of genome assembly.

## Results and discussion

### Computational complexity of the problem

The problem of ordering and orientation of genomic scaffolds is well known to be NP-hard [2,24]. A genomic map provides information that implies the relative placement of scaffolds. When either the genomic maps or the scaffold assemblies contain errors, or there are conflicts between the multiple input maps, the problem of finding the optimal scaffold ordering and orientations that satisfy the most constraints becomes intractable [2,24]. Studies on scaffolding based on read pairs largely rely on heuristics [1,2,24]. In the ALLMAPS implementation, we chose to use Genetic Algorithm (GA) instead of some other heuristics such as local search, hill climbing, and greedy strategy to avoid getting stuck in local optima [25,26]. Other related problems such as consensus mapping has adopted similar evolutionary strategy [14].

### Simulation

To assess the accuracy of ALLMAPS, we simulated a set of scaffolds with random orientations along the whole genome of *Medicago truncatula*, as the ‘truth’ to be evaluated against. The published Medicago genome has eight chromosomes, with 366 Mb in total length. Each of the eight chromosomes represents a typical complex eukaryotic chromosome. In order to have a length profile similar to real data, the scaffold length distribution of these simulated scaffolds was modeled from a preliminary assembly used in Mt4.0 (20,591 sequences in total, largest 346 Kb, scaffold N50 = 46 Kb) [8]. A total of 8,000 genetic markers were generated from the Medicago genome following a uniform distribution and two-thirds of the markers were assigned to each genetic map following a random distribution along the genome. The marker density of each genetic map is 13.7 markers per Mb. To evaluate the accuracy of our program, we used the ratio between the longest monotonic subsequence (*LMS*) and the total number of markers as an indication of colinearity between the ALLMAPS answer and ‘truth’. This ratio varies between 0 and 1 - an accuracy of 1 means all markers are in perfect colinearity between the scaffold order and the input maps.

**Figure 1:**
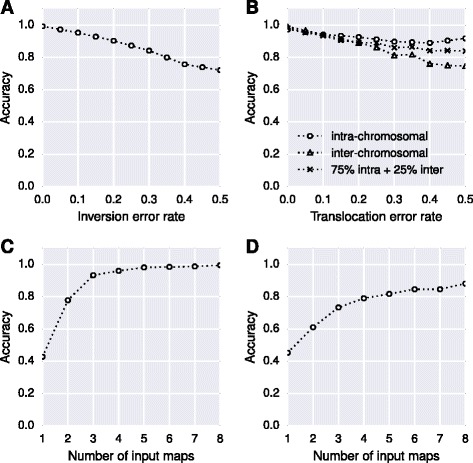
**Evaluation through simulated datasets to test the robustness of ALLMAPS. (A)** Inversion errors (*P*_*inv*_) against accuracy; **(B)** translocation errors (*P*_*trans*_) against accuracy; **(C)** number of input maps (where one map contained 20% errors) against accuracy; **(D)** number of input maps (where all maps contained 20% errors) against accuracy. For translocation error analyses in this study, 75% translocated markers are within chromosomes and 25% translocated markers are between chromosomes.

To show how ALLMAPS responds to errors in the maps, we implanted a few types of errors into the simulated maps. The error types include inversion and translocation errors, two common errors in constructing genetic maps. Inversion refers to a chromosome arrangement where a genomic segment is reversed from end to end compared to the reference. Translocation refers to the type of rearrangement of parts moved to non-homologous chromosomes. For inversion errors, the error probability (*P*_*inv*_) was defined as number of markers involved in inversion divided by total number of markers for each chromosome. (*P*_*inv*_) of 0.5 means half of the markers on the chromosome were involved in an inversion error, which is the most extreme error case. The translocation error probability (*P*_*trans*_) was defined as the number of markers involved in translocation within a chromosome (intra-chromosomal) or between chromosomes (inter-chromosomal) divided by the total number of markers. For translocation error analyses in our study, 75% translocated markers are intra-chromosomal errors and another 25% are inter-chromosomal errors. For each of these two error analyses (inversion and translocation errors), ALLMAPS took two genetic maps as input, only one of which contained errors. Our results revealed that the accuracy of ALLMAPS was affected by both of the error probability, with inversion error has a slightly larger impact on the accuracy than translocation (Figure 1A). Inter-chromosomal translocation error has a larger impact than intra-chromosomal translocations on the accuracy, since the error affects a much earlier stage (linkage group clustering) during the ALLMAPS pipeline (Figure 1B).

Next, we investigated the relationship between the accuracy and the numbers of genetic maps used for scaffolding. In this case, we introduced moderate level of errors (*P*_*inv*_ = 0.2 and *P*_*trans*_ = 0.2) into one genetic map (while not introducing errors into the other maps) and allowed ALLMAPS to take from one to eight genetic maps as input. As expected, ALLMAPS’s performance was improved as the number of available genetic maps increased (Figure 1C). The trend shows that big improvement of accuracy between one and two maps, and between two and three maps or more. The curve flattens when there are three input maps, suggesting that it is beneficial to have at least three maps for error correction. There is still noticeable improvement using more than three maps in this simulated study but the accuracy quickly approaches 1 when more maps are used as input. For a much stronger test, we introduced noise to all input maps at 20% errors (*P*_*inv*_ = 0.2 and *P*_*trans*_ = 0.2) and allowed ALLMAPS to take from one to eight genetic maps. When all input maps contained errors, ALLMAPS require more maps to be able to deduce the correct scaffold ordering, with accuracy approaching 0.9 only when eight maps were used (Figure 1D).

### Application in construction of yellow catfish chromosomal assemblies

While simulated data revealed some basic properties of ALLMAPS, we applied ALLMAPS to a real-world genome project: yellow catfish (see Data availability). Genomic scaffolds were generated as part of the ongoing project to sequence the yellow catfish genome. We generated 100 bp paired end and mate pair reads (SRA accession: SRP050322) on an Illumina HiSeq 2000, which were assembled into scaffolds using ALLPATHS-LG [27]. The yellow catfish project also generated genetic maps based on the progeny of a bi-parental cross between two individual heterozygous yellow catfish. Our goal was to use ALLMAPS to anchor scaffolds from a draft genome assembly of yellow catfish into pseudomolecules using these genetic maps. The scaffold assembly of yellow catfish consists of 9,224 scaffolds (*N50* = 1 Mb) that comprise 718 Mb. The 161 scaffolds with a length of greater than 1 Mb (‘*N50* scaffolds’) are our main focus in the anchoring process. SNPs derived from tGBS sequencing [28] were categorized into two subgroups basing on their segregation ratios in the population and parental genotypes. Based on a back-cross model [15], two genetic maps were constructed from each of the subgroups using R/QTL [17], that we called BCMale and BCFemale. Using the same subgroups of markers, but running a different mapping software (JOINMAP [19]), we obtained two additional maps that were called JMMale and JMFemale. We used all four maps (BCMale, BCFemale, JMMale, JMFemale) to order and orient scaffolds into pseudomolecules. We will show that creating two sets of maps with different software from the same data could improve the assembly.

We were able to anchor a total of 581 Mb by using four genetic maps simultaneously, or approximately 50 Mb more sequences anchored than any single map alone (Table 1). Overall, ALLMAPS anchored 81% of the bases onto the 26 chromosomes of yellow catfish. In particular, all but one of the 162 *N50* scaffolds were anchored to the chromosomes. The orientations of scaffolds with only a single marker cannot be determined without additional evidence and these are labeled with unknown orientations in the AGP output. All but eight input markers were placed onto the chromosomes (Table 1). These eight markers and the associated scaffolds which failed to be incorporated into the final pseudomolecule assembly were excluded because of their ambiguous placement on multiple chromosomes in different genetic maps. These unplaced scaffolds may be chimeric or the tGBS markers on these scaffolds may be located in repetitive regions. The majority of unplaced sequences are small fragmented scaffolds that contain no markers in any of the genetic maps. Indeed, the remaining 8,282 scaffolds that do not have chromosome assignments have average size of 17 Kb. In addition, they contain an average of 22% ambiguous bases (N), suggesting that these scaffolds might represent repetitive portions of the genome that are difficult to assemble into large chunks.

**Table 1:** **Summary statistics for each of the four component maps (BCFemale, BCMale, JMFemale, JMMale, with equal weights) and final consensus anchoring (‘Anchored’) in the yellow catfish study**

	**BCFemale**	**BCMale**	**JMFemale**	**JMMale**	**Anchored**	**Unplaced**
Linkage groups	26	27	26	27	26	n.a.
Markers (unique)	2,507	2,442	2,495	2,434	4,941	8
Markers per Mb	4.8	4.6	4.8	4.6	8.5	0.1
*N50* scaffolds	158	160	158	160	161	1
Scaffolds	679	709	673	707	942	8,282
Scaffolds with 1 marker	291	302	286	303	325	0
Scaffolds with 2 markers	109	140	109	138	168	2
Scaffolds with 3 markers	63	65	63	64	92	0
Scaffolds with ≥4 markers	216	202	215	202	357	1
Total bases	524,479,992	534,215,264	523,736,261	533,964,013	580,865,792	138,224,118
(Percent of genome)	(72.9%)	(74.3%)	(72.8%)	(74.3%)	(80.8%)	(19.2%)

Scaffolds with no markers, or ambiguous placements, are separately counted (‘Unplaced’). The marker density for the anchored and unplaced scaffolds represent the sum of unique markers from all input datasets.

### Robust integration of multiple maps

**Figure 2:**
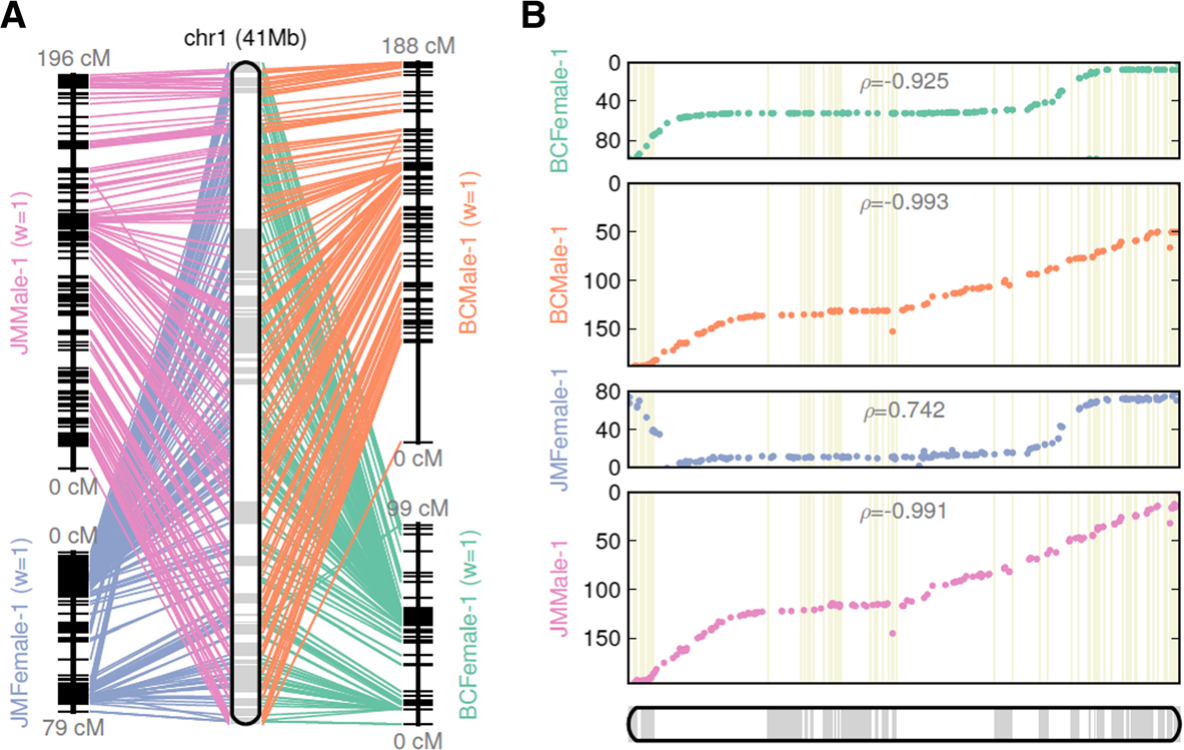
**Pseudochromosome 1 of yellow catfish genome, reconstructed from four input maps - BCFemale, BCMale, JMFemale, JMMale, with equal weights of 1. (A)** CMAP-style presentation with lines connecting the physical positions on the reconstructed chromosome and the map positions. **(B)** Set of four scatter plots, with dots representing the physical position on the chromosome (*x*-axis) versus the map location (*y*-axis). Adjacent scaffolds within the reconstructed chromosome are shown as boxes with alternating shades, marking the boundaries of the component scaffolds. The ρ-value on each scatter plot measures the Pearson correlation coefficient, with values in the range of -1 to 1 (values closer to -1 and 1 indicate near-perfect colinearity).

While we show that the sequence anchor rate can be greatly improved with multiple maps compared to using single map, the integration of multiple maps can also result in major improvements in assembly accuracy. To illustrate the significance of the use of multiple maps, we show one representative chromosome (chr1) in the yellow catfish genome assembly (Figure 2). The four input maps are complementary to one another in scaffold anchoring in two important ways. First, there are drastic differences in recombination rates between the maps. The ‘slopes’ in the scatter plots reflect changes in the ratio between the physical (*x*-axis) to genetic (*y*-axis) distance, which is equivalent to the recombination rate. This drastic difference in recombination rate is evident as sudden change of slopes in the female maps (BCFemale, JMFemale) in the telomeric end of chr1 (Figure 2), which was also previously observed in rainbow trout [29]. Low recombination rates can lead to ambiguous orderings between scaffolds that are located within the same ‘recombination bin’. By considering the maps derived from both genders, ALLMAPS created a map with better resolution than each single map alone (Figure 2). Second, we were able to fix the errors present in any one map through a voting procedure (majority rules). For example, JMFemale-2 contained an erroneous inversion at the short arm of chr1 that is absent from the other three maps (Figure 2), which appears to be a problem with JOINMAP in that particular region of the chromosome in the JMFemale map. ALLMAPS was able to correctly order and orient these scaffolds that provide the most concordance between the maps constructed from different genders.

When equal weights are given for each of the input maps, the final order is a majority-rule consensus among all input maps. However, users can also place a weight on each map to reflect their preference or confidence based on independent evaluations of accuracy. A useful usage pattern then emerges: the users can modify weights for the input maps, examine the summary report and diagnostic plots, and then iterate with different sets of weights. This is a supervised usage of ALLMAPS, as opposed to the default behavior of treating each map with the same weight (weight = 1). The map weights affect important aspects of the ALLMAPS algorithm, particularly on how the conflicts are resolved between maps. The weights affect to which chromosome a scaffold may anchor, as well as the final order and orientations between linked scaffolds. Naturally, the final scaffold configuration will be biased towards the map with the highest assigned weight.

### Extension to other types of genomic maps

**Figure 3:**
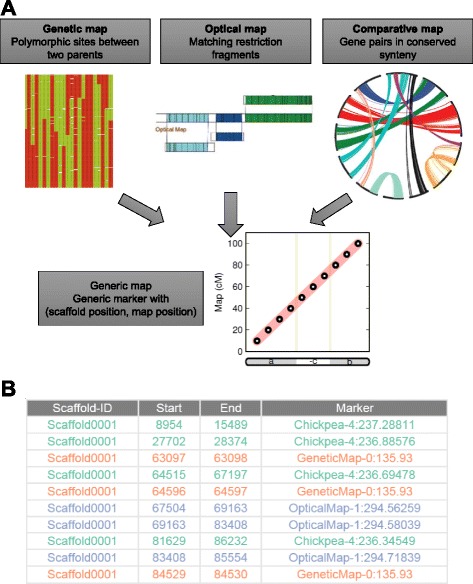
**Integration of various mapping evidence inside the ALLMAPS framework. (A)** Various map types converted to a ‘coordinate-based’ generic marker type that allows universal treatment. **(B)** Example from Medicago to demonstrate ALLMAPS input BED format. Markers derived from genetic map, optical map, and comparative map are highlighted in different colors.

After applying ALLMAPS to multiple genetic maps, we further highlight ALLMAPS’s ability to go beyond genetic maps to use other types of genomic maps. A combination of several independent lines of evidence will further complement each other in different regions of the genome. There are specialized methods for handling different mapping data individually, including tools for optical map alignment [9,12] and synteny-guided assembly [4,13], but these methods were never combined within the same framework to exploit multiple maps. One obstacle is that other genomic maps may require some data transformation before being imported into ALLMAPS. We note that a generalized form of genomic maps is constituted by markers represented as (*x, y*) - each marker having a coordinate on the genomic scaffolds (*x*) and another coordinate on the map (*y*), respectively. As long as the map can be converted into a list of abstract ‘markers’ carrying these two coordinates (in a standard BED format), they can be easily integrated in a unified framework (Figure 3). Details of this format and tools to transform data from optical map alignments and synteny alignments are available with the distribution of ALLMAPS: [30].

To test the performance of ALLMAPS on utilizing optical mapping data, we used the publicly available budgerigar dataset, which was also used in the Assemblathon 2 competition [31,32]. The budgerigar v6.3 scaffolds were aligned to the optical maps, based on which the mega-scaffolds were constructed and published [31,32]. Among a total of 49 suggested joins, ALLMAPS was able to recover 44 scaffold links (90%) with correct ordering and orientations. The remaining five links were manually inspected and were found not supported by optical mapping data (that is, in different optical map contigs). We suggest that these five discrepancies between ALLMAPS result and the published assembly might be due to other linking evidences such as read pairs that were not accessible by ALLMAPS.

To illustrate the full benefits of using multiple mapping technologies, we tested ALLMAPS on data derived from the genome of *Medicago truncatula*, where different types of genomic maps are publicly available (see Data availability). The Medicago genome project has developed a suite of maps that were incorporated into the Mt4.0 genome assembly [8]. In particular, an optical map is available (Genbank nucleotide database accessions MAP_000013 to MAP_000020). Optical mapping is a technique to build high-resolution restriction endonuclease maps and has been widely used in reconstructing chromosomal assemblies [8-10]. Here, we treated matching restriction fragments between the optical map and *in silico* restriction fragment from the scaffolds as ‘markers’ with each marker having two coordinates for its position on a scaffold and on the optical map (Figure 3A). The second map is a genetic map constructed from a double-haploid population. For the third map, we use a comparative map between chickpea and Medicago. Chickpea has a high quality genome assembly and can be used to anchor Medicago scaffolds due to their evolutionary relatedness [8,33]. Synteny evidence can be utilized by treating syntenic gene pairs between the two species as ‘markers’, and transforming the data into the appropriate form for ALLMAPS (Figure 3B).

Different weights were assigned to each map in this integration, reflecting our relative confidence to each of the map: weight of 3 for the optical map, 2 for the genetic map, and 1 for the chickpea comparative map. In particular, the comparative synteny evidence should always be used with extra caution since the disruption of colinearity can be due to real evolutionary events such as chromosomal rearrangements, fusions, fissions, and translocations. Assigning the lowest weight to the comparative map ensures that it would never be considered when in conflict with the other two maps.

Results of ALLMAPS applied to the Medicago scaffolds and three input maps are summarized in Table 2. Overall, ALLMAPS anchored 384 Mb of scaffold sequences onto the eight chromosomes, more than the anchor rate based on any single map alone, and matched the anchor rate in the published Mt4.0 assembly that is a product of intensive manual curation [8]. All *N50* scaffolds in Medicago assembly were anchored onto the chromosomes. The unplaced scaffolds show an average of 1.6 markers per Mb, much lower than the marker density on the anchored scaffolds (Table 2).

**Table 2:** **Summary statistics for three component maps and final consensus anchoring (‘Anchored’) in the Medicago study**

	**OpticalMap (w = 3)**	**GeneticMap (w = 2)**	**Chickpea (w = 1)**	**Anchored**	**Unplaced**
Linkage groups	8	12	98	8	n.a.
Markers (unique)	25,491	2,125	16,275	43,833	58
Markers per Mb	70.2	5.8	43.2	114.0	1.6
*N50* scaffolds	23	23	23	23	0
Scaffolds	123	200	229	284	4,522
Scaffolds with 1 marker	0	68	53	84	2
Scaffolds with 2 markers	0	21	26	26	1
Scaffolds with 3 markers	0	22	10	15	1
Scaffolds with ≥=4 markers	123	89	140	159	8
Total bases	363,160,642	363,369,814	376,760,724	384,460,302	36,076,813
(Percent of genome)	(86.4%)	(86.4%)	(89.6%)	(91.4%)	(8.6%)

Scaffolds with no markers, or ambiguous placements, are separately counted (‘Unplaced’). The marker density for the anchored and unplaced scaffolds represent the sum of unique markers from all input datasets.

**Figure 4:**
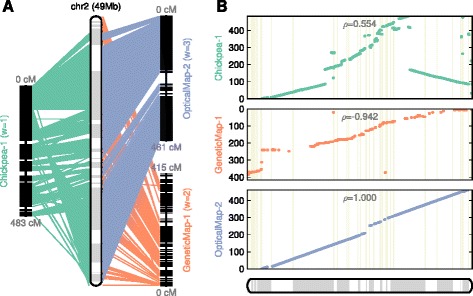
**Pseudochromosome 1 of** ***Medicago truncatula*** **genome, reconstructed from three input maps - optical map with weight of 3, genetic map with weight of 2 and chickpea synteny with weight of 1, to show the capability of ALLMAPS to integrate heterogeneous map types using arbitrary weights. (A)** CMAP-style presentation with connecting lines as matching markers. **(B)** Set of three scatter plots, with each dot representing the physical position on chromosome (*x*-axis) versus the map location (*y*-axis). The cM distance along optical map and chickpea comparative map are scaled by 1 cM = 100,000 bp for illustration purposes. This linear transformation does not affect the computation of scaffold configuration. Note that the map based on synteny to chickpea, a divergent species from Medicago, shows the highest level of discordance.

We found that the optical map has poor support in centromeric regions, probably due to an abundance of tandem repeats, making the restriction fragment pattern less unique, which in turn reduces the align-ability of optical map data in those regions (Figure 4). In contrast, both the genetic map and the chickpea comparative map contain a number of supporting markers in those regions. The genetic map and the chickpea synteny map complement the optical map, providing the scaffold tiling across the centromeric region of chr2 (Figure 4). Both optical map and genetic maps are largely concordant in providing high levels of colinearity to the reconstructed chromosome assembly, while colinearity to the chickpea comparative map (Chickpea-1) is disrupted by an apparent translocation, likely due to the evolutionary divergence of genome structures between Medicago and chickpea (Figure 4). The concordance between the final reconstructed chromosome and each of the three maps matches the gradation of assigned weights (optical map > genetic map > chickpea synteny). The Spearman correlation coefficient is the highest (1.000; completely colinear) for optical map, medium (-0.942) for genetic map, and lowest (0.554) for chickpea synteny due to the big translocation.

Different mapping techniques tend to show different marker density characteristics and therefore likely complement each other in different regions along the chromosomes. Some genome projects that have had access to a multitude of maps were able to identify major discrepancies after checking two or more lines of evidence [10,21,33]. Our ALLMAPS framework is applicable to all coordinate-based maps or maps that can be converted into coordinate-based through transformation, therefore substantially reducing the overhead for finding the correct assembly tools to handle the plethora of mapping evidence. Using ALLMAPS to consolidate different maps substantially increases the completeness as well as the accuracy of the scaffold anchoring, generating very high quality draft assemblies.

When there is an unfortunate shortage of available genomic maps, for example, in ‘orphan’ species where there is little research investment in the past, we can still create consensus chromosomal assemblies based on comparative maps against multiple, closely-related genomes as a collection of ‘references’ [24]. Each related genome may be assigned a weight, reflecting their evolutionary distance to the organism of interest. When only synteny data from multiple species are used, the assembled sequences by ALLMAPS might resemble an ‘ancestral’ chromosome arrangement, an idea exploited during the reconstruction of the Black Death agent *Yersinia* genome [34]. Most existing tools for comparative assembly developed to date can only exploit a single ‘reference’ to assemble against [4,13]. Through the creative use of ALLMAPS, we can greatly expand the types of evidences that can be applied to a genome assembly even in situations where mapping evidence is scarce.

### Factors affecting the performance of ALLMAPS

**Figure 5:**
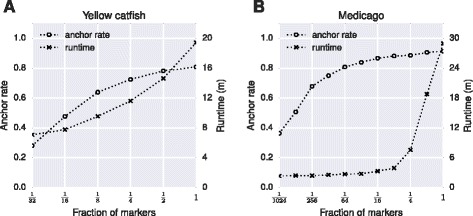
**Effect of marker density on the performance of ALLMAPS based on resampling of real data.** The scaffold anchor rates and program running times were assessed based on the sub-samples of increasing sizes for **(A)** yellow catfish and **(B)** Medicago datasets.

Marker density is a key factor contributing to both accuracy and scaffold anchor rate of ALLMAPS. More markers would require more computational power to resolve, but fewer markers could decrease the usefulness of the maps in terms of scaffold anchoring. We carried out resampling studies for the yellow catfish and Medicago datasets to demonstrate the influence of marker density on the performance (Figure 5). The yellow catfish contains a relatively low marker density (Table 1), and the scaffold anchor rate quickly dropped when only a fraction of all data was used (Figure 5A). However, the Medicago data still showed nearly 80% anchor rate even when 1/64 of the markers were retained (Figure 5B), due to the large number of markers derived from optical map and synteny map (Table 2). The running time of ALLMAPS generally shows linear relationship to the number of input markers, but could increase substantially for ultra-dense datasets (Figure 5B).

Recently, dense genetic maps can be generated with inexpensive genotyping technology such as GBS and RAD, and most published NGS genomes projects have already adopted these high-density genetic mapping protocols [15,16,20]. Optical maps often contain more markers because markers are derived from restriction fragments, which are often abundant in a large genome (for example, six-base cutter cuts every 4 Kb, on average). The density of markers in a comparative map is determined by the number of genes in conserved synteny blocks and divergence between the genomes in comparison [23].

Errors during the genome assembly and map making protocols could potentially propagate into the ALLMAPS assembly. The starting quality of the genome assembly based on read overlaps and read pairing is therefore crucial to the performance of ALLMAPS. A highly fragmented assembly could produce sub-optimal or erroneous results. In various mapping protocols, there are technological problems like simple genotyping errors that could lead to bad maps with missing or translocated segments. ALLMAPS warns about the chimeric scaffolds with mappings to multiple linkage groups, but relies on the user to provide relatively clean maps and scaffold assemblies to achieve the best results.

Specific genome structural features might cause additional problems during assembly, including chromosomal inversion, translocation, and segmental duplication. Map data that do not reflect genomic arrangement in the reference individual would cause incongruities among the input maps (for example, synteny data from species with rearrangements, or genetic maps from individuals with structural changes relative to the mapping parents). The errors in the input maps could potentially have a common biological cause. For example, segmental duplication disrupts genome assemblies reconstructed from short reads and might similarly affect various types of genomic maps [35]. If the segments are divergent enough to be properly assembled, identifying markers and map based ordering would work as well for the duplicated segments as for other sequences. However, if the duplicate regions are collapsed into single scaffolds by the genome assemblers, ALLMAPS would not be able to separate these misassembled regions.

Despite various technical and biological complexities associated with making accurate assemblies and maps, ALLMAPS is designed to incorporate different types of map data including genetic, optical, and synteny data which are expected to have very different unlinked error profiles. For example, while the repetitive sequences or copy number variations (CNVs) might affect the optical map (generating similar restriction fragment patterns) and to a lesser degree also affect the synteny map, the genetic maps are less likely affected by the errors derived from repeats or CNVs. Repetitive sequences and CNVs produce distinctive segregation ratios and would be removed prior to genetic map construction. Conversely, genetic maps do not provide coverage for the non-recombinogenic regions like centromeres and sex chromosomes. This weakness of genetic maps could otherwise be remedied by incorporating other types of maps that are not based on genetic recombination.

## Conclusions

We show that ALLMAPS is capable of integrating several lines of mapping evidences to guide the assembly of genomic scaffolds. A key feature of ALLMAPS is its ability to integrate information from multiple maps on the basis of a well-defined objective function to maximize the colinearity score. Each map can be assigned a weight, allowing flexible tuning based on users’ confidence on each input map. ALLMAPS identifies the consensus from several maps, resolves conflicts based on user assigned weights and consolidates these results into a highly consistent scaffold ordering given the available mapping evidence. ALLMAPS can incorporate other types of maps including physical maps, optical maps and comparative maps as well, thus offering a one-stop shop for robust integration of scaffold linkage evidence from a variety of popular mapping technologies, resulting in higher coverage, more accurate, and more replicable chromosome-level genome assemblies.

## Methods

### ALLMAPS objective and algorithm overview

**Figure 6:**
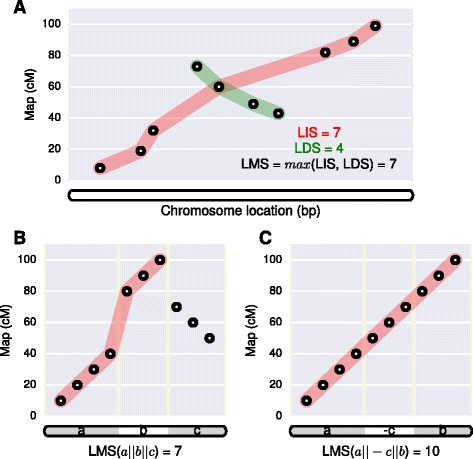
**Longest monotonic subsequence (** ***LMS*** **) objective function visualized on a scatter plot.** Circles on the scatter plot represent ‘markers’ that are indicated by their base positions on the scaffolds and genetic positions on the map. **(A)** *LMS* calculated as the larger value of longest increasing subsequence (*LIS*) and longest decreasing subsequence (*LDS*). **(B)** Example scaffold configuration that is not optimal when evaluated with *LMS* function. **(C)** Optimal scaffold configuration for the same three scaffolds.

The goal of ALLMAPS is to find the order and orientation (or in combination the ‘configuration’) of genome sequence scaffolds that maximizes the ‘colinearity’ of various chromosomal markers. A marker implies both a physical location on a scaffold and a map location (Figure 6). In the context of genetic mapping, the map location is indicated by cumulative genetic distance, measured in centiMorgans (cM). Later, possible extensions to other types of maps will be discussed, however the core algorithm remains constant regardless of the type of mapping data being employed.

The colinearity between the array of physical locations on the DNA sequence and the array of map locations can be assessed, given a specific configuration of all scaffolds. Assuming the physical locations are already sorted, we can estimate the colinearity via the length of its longest increasing subsequence within the map locations. An increasing subsequence refers to a subsequence in which the elements are in sorted order, from lowest to highest. In this context, the length of a subsequence is equal to the number of markers. Because the polarity of the linkage group was arbitrarily determined during map construction, we may look for either the longest increasing subsequence (*LIS*) or longest decreasing subsequence (*LDS*). Without loss of generality, we define the ‘longest monotonic subsequence’ (*LMS*) as the larger of *LIS* and *LDS* to indicate the degree of colinearity, irrespective of the polarity of the map (Figure 6A).

The ‘scaffold anchoring problem’ is therefore to find a scaffold configuration that maximizes the sum of *LMS* to the input maps, each with an assigned weight. The significance of weights will be discussed in further detail later. We introduce the following set of notations for a more formal description of the scaffold anchoring problem:

- *S* - Set of scaffolds
- *G* - Set of maps
- *w*_*j*_, where *j* ϵ *G* - Weight for the *j*-th map
- *M*_*ij*_, where *i* ϵ *S*, *j* ϵ *G* - Ordered set of markers on *i*-th scaffold and *j*-th map. Each marker carries two positions - physical position on the scaffold and genetic position on the map. Within the set of *M*_*ij*_, markers are ordered according to increasing base pair positions on the scaffold. With a leading ‘minus’ sign, - *M*_*ij*_ indicates the same set of markers ordered according to decreasing base pair positions on the scaffold
- *LMS* (*M*_*ij*_) - Longest monotonic sequence of genetic positions among the ordered markers in *M*_*ij*_. We note that this directly measures the colinearity between the physical positions and the genetic positions.

We introduce the notation *a || b* to show a configuration where scaffold *a* and *b* are adjacent, and use - *a* to indicate scaffold *a* is anchored in the reverse orientation. We can calculate the colinearity of markers using *LMS* score for any given scaffold configuration. Our goal, then, is to find the scaffold configuration that yields the highest *LMS* score possible. An illustrative example on how to evaluate the scaffold configuration against a single map is given in Figure 6B and C. In the case of more than one maps, we look for the scaffold configuration that maximizes colinearity across all maps. As an example, given the configuration *a* || - *b* || *c*, we can calculate the *L* score according to the following objective function, which is the weighted sum of *LMS* across multiple maps:

$$ L\left(a\left|\right|-b\left|\right|c\right)={\displaystyle \sum_{j\in G}{w}_j \cdot p\ LMS\left({M}_{aj}\left|\right|-{M}_{bj}\left|\right|{M}_{cj}\right)} $$

**Figure 7:**
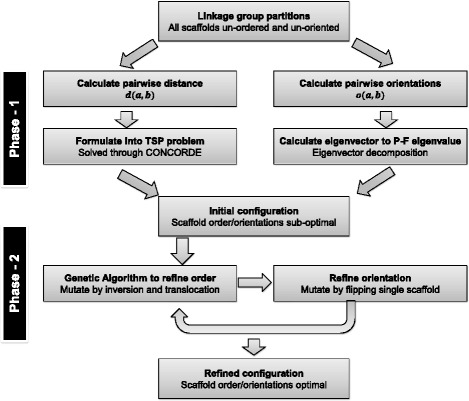
**Illustration of the major steps in ALLMAPS algorithm.** The ALLMAPS method contains two phases to calculate an initial configuration of scaffolds followed by iterative refinement.

To compute the optimal scaffold configuration is to maximize the score of *L* over all possible configurations, which is an optimization problem. Since there can be exponential number of possible scaffold configurations, an efficient search strategy is required. In ALLMAPS, the optimization of *L* is conducted in two phases, detailed below and illustrated in Figure 7. In Phase 1, we compute an approximate scaffold configuration to speed up the computation. In Phase 2, we use a genetic algorithm to refine the scaffold configuration and incrementally improve on the final score *L*. The computation of the initial orientation and ordering is very fast in Phase 1, which can cut down the running time required for convergence in the more accurate, but slower, Phase 2.

### Clustering of homologous linkage groups

Prior to computing the scaffold configuration in the two phases, we divide the whole problem into several sub-problems, with each sub-problem representing a single chromosome that can be solved independently. We first define the ‘pivot map’ as the map with the largest weight, or the map that occurs first in the user input in case of ties between weights. The pivot map determines the number of chromosomes to appear in the assembly. Consequently, the pivot map should ideally contain the same number of linkage groups as the number of chromosomes in the target organism. We assign ‘homologous’ linkage groups from all input maps into separate clusters, with each cluster seeded by a single linkage group from the pivot map. Each linkage group from a non-pivot map will be assigned to the cluster with which it shared the most number of scaffolds. Once the clustering is completed, each scaffold is assigned to a single best cluster based on the number of markers. To reduce ambiguity, chimeric scaffolds that map well to two or more linkage group clusters should preferably be split prior to the execution of ALLMAPS. To determine whether a scaffold is chimeric, ALLMAPS counts the sum of markers mapped to each linkage group cluster, weighted across all input maps. ALLMAPS alerts the user to the chimeric cases with equally good mappings to two or more linkage clusters and skips them during its computation. As a final step in the preprocessing step, we apply Iglewicz and Hoaglin’s outlier test to remove markers that appear to have erratic genetic positions [36]. These markers are most likely due to genotyping errors or other artifacts when the map was constructed.

### Phase 1: Generate an initial scaffold order and orientation

This step is the first phase in finding an optimal configuration of scaffolds. There are two aspects in this phase, finding an optimal ordering and finding the optimal orientations of scaffolds. We solve these two problems separately.

First, to get the orientation for each scaffold, we compute the relative orientation between all pairs of scaffolds. We infer the relative orientation *o* (*a*, *b*) between two scaffolds *a* and *b* by calculating the difference of colinearity scores between ‘same-orientation’ (*a* || *b*) and ‘opposite-orientation’ (*a* || - *b*) configurations:

$$ o\left(a,b\right)={\displaystyle \sum_{j\in G}{w}_j \cdot p\ \left[LMS\left(a\left|\right|b\right)-LMS\left(a\left|\right| - b\right)\right]} $$

We note that the sign of *o* (*a*, *b*) determines the relative orientation between scaffold *a* and *b*. A positive score indicates that scaffold *a* and *b* are of the same orientation, a negative score indicates opposite orientation, and zero score indicates undetermined orientations. We store the relative scaffold orientations between all pairs of scaffolds in a square matrix *M*, with number of rows equal to the number of scaffolds. We then compute the eigenvector *y* corresponding to the largest eigenvalue of *M*, also known as the Perron-Frobenius eigenvalue. The signs of the components in *y* provide an approximate solution to flipping the scaffolds to achieve the highest consistency given all pairwise orientations.

To calculate the ordering among the scaffolds, we used an objective function that’s different from the final *L* score but nonetheless highly correlated. We define the pairwise distance *d* (*a*, *b*) between two scaffolds *a* and *b* as the distance between the closest markers between the two scaffolds:

$$ d\left(a,b\right)={\displaystyle \sum_{j\in G}{w}_j \cdot p\ \underset{x\in {M}_{aj},y\in {M}_{bj}}{ \min}\left( abs\left(x-y\right)\right)} $$

With all pairwise scaffold distances calculated, we seek the ordering that yields the least sum of distances between adjacent scaffolds. This problem is then analogous to the famous ‘traveling salesman problem’ (TSP). We calculate the ordering using CONCORDE, currently considered to be the best TSP solver so far [37]. CONCORDE has been applied to solve a variety of bioinformatics problem including radiation mapping [38] and prediction of protein functions [39]. CONCORDE runs quickly within seconds in all real-world scaffold anchoring problems that we have tested thus far, making it an ideal solver for an approximate solution in Phase 1.

The initial ordering based on the minimization of the inter-scaffold distance across multiple maps is often close to the final solution that maximizes colinearity, suggesting that these two objectives are largely correlated. However, TSP can still generate sub-optimal solutions that require further tuning. For example, the maps may still be very noisy so that even the markers within a single scaffold may not be collinear, which are then likely to skew the distance calculation. However, since our goal in Phase 1 is to simply cut down the total running time by minimizing the search space for Phase 2, this does not have to yield the exact final solution.

### Phase 2: Refine order and orientation using a genetic algorithm

The initial scaffold configuration calculated in Phase 1 is close to the final solution, but is sometimes sub-optimal in the *L* score. In Phase 2, we apply a Genetic Algorithm (GA) to further refine the order and orientation of the scaffolds. Indeed, we could skip Phase 1 and directly run GA to maximize the *L* score from the start, but formulating the problem as TSP to compute an initial solution speeds up the GA in Phase 2 so that ALLMAPS can quickly converge on a final solution.

A standard GA strategy operates on a population of ‘individuals’, where each individual represents one possible solution - or in our problem, one possible scaffold configuration. We apply ‘mutation’ and ‘crossover’ operations to introduce changes to the current pool of individuals, while each individual is evaluated with respect to their ‘fitness’ (the *L* score) after the change. Individuals with high fitness scores are preferentially retained (elitist selection) in each generation.

At the start of GA, all individuals are instantiated with the configuration we computed in Phase 1. We then apply two types of mutations at each generation: ‘inversion’ which randomly selects two points in each solution and reverses the order of the scaffolds in between; ‘insertion’ which randomly translocates a scaffold and inserts it next to another randomly selected scaffold. These two mutation operators represent both large-scale and small-scale changes. For crossover operator, we use the ‘Partially Mapped Crossover’ (PMX) function that was shown to speed up convergence [40]. The overall GA scheme is configured with mutation and crossover probability of 0.2 and 0.7, respectively, which were selected to offer a rapid convergence out of a range of test values. The population size is set at 100, and is allowed to evolve until there is no change of best solution in the last 1,000 generations as convergence criteria. These options can be adjusted at runtime. Generations beyond 1,000 can further ensure that the solution converges to the optimal, but also lead to longer running time. To further boost performance, we used parallel processing during the fitness evaluation which is the most time-consuming step during GA [40].

After the order is changed in one round of GA, we iterate through the scaffolds and check if flipping of any scaffold would increase in the final score. The scaffold is then flipped if an improvement is possible. The order and orientation of scaffolds are two intertwined problems with the results of one affecting the other throughout the computation. If the orientations are changed, then the order among the scaffolds needs to be re-optimized. Similarly, if the order is changed, then the orientations need to be re-optimized. Therefore, several rounds of refinement are sometimes necessary before converging on a final solution. Each round is consisted of optimization of scaffold order followed by optimization of the orientations, until no further improvement in score can be made (Figure 7).

### Genome release, summary statistics, and visualization

After computing the scaffold configuration, ALLMAPS proceeds to build the genome assembly. The scaffolds are concatenated together according to the computed order and orientations. Pseudomolecule sequences are constructed with 100Ns (configurable) padded between the scaffolds to represent inter-scaffold gaps. Three outputs - FASTA, AGP, and CHAIN files are the key outputs of the chromosomal assemblies. The FASTA file contains the nucleotides of the pseudomolecules and unanchored sequences. The AGP file is a standard file format to describe assembly of the pseudomolecules from the scaffolds, specifying both the order and orientations. The header of the AGP file contains metadata of the operation, tracking input files and parameters when running ALLMAPS. The CHAIN file, when used in conjunction with the UCSC ‘liftOver’ tool, allows easy conversion from scaffold coordinates to final chromosome-level coordinates [41]. This can be very useful, for example, when gene features were initially predicted on the scaffolds but need to be transferred onto the chromosomes. A byproduct of the liftOver procedure is a consensus map constructed from all input maps. This consensus map is constructed via a common physical order along the reconstructed chromosomes and therefore different from other implementations of consensus mapping [14,42].

A summary report is provided to the user during the genome build, with important statistics such as the number of scaffolds anchored, the number of big (*N50*) scaffolds anchored, total number of markers included in the final assembly and total length of sequences. These summary statistics can be useful tools to compare the efficacy of ALLMAPS before and after anchoring. We offer two popular ways of visualizing alignments between the markers and reconstructed chromosomes. The first visualization is the ‘side-by-side’ (also known as ‘parallel coordinates’) alignments between chromosomes and the linkage groups, with connecting lines showing the location of the markers (Figure 2A). This type of plot is helpful to reveal conflicting markers as crossing lines, which is also used in CMAP [43]. The second visualization is scatter plot, where the coordinates of the dots represent the physical locations and the map locations of the markers, for each input map (Figure 2B). The scatter plots are a good visualization for illustrating the monotonic trend as well as revealing breaks in colinearity. Through visual inspection of these plots, it is possible to quickly assess the concordance between the final ordering of scaffolds against each of the input maps used in the assembly.

### Gap size estimation

**Figure 8:**
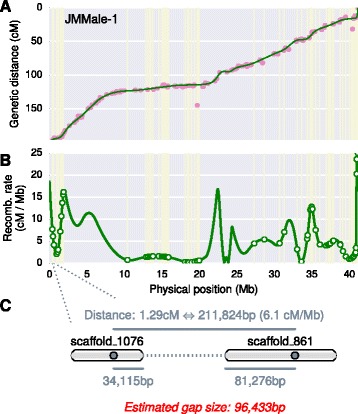
**Algorithm for estimation of inter-scaffold gap lengths. (A)** Scatter plot, with dots showing the physical position on the chromosome versus the genetic position. Vertical lines mark the boundaries of the component scaffolds. Cubic spline is used to generate interpolation of genetic distance along the chromosome. **(B)** Recombination rates, measured in centiMorgans per Mb (cM/Mb), are estimated by taking the derivative of the cubic spline. Circles represent the locations of the inter-scaffolds gaps. **(C)** An example of the size estimation of one gap between two adjacent scaffolds.

All inter-scaffold gaps were configured to be a fixed size of default 100Ns during the genome release. Some users may wish to get more refined size estimates of the gaps. Accurate inference of gap size is dependent on the conversion between the map distances to the physical distances. In the case of genetic maps, this conversion ratio is known as the recombination rate, commonly measured in centiMorgan per Mb (cM/Mb). The recombination rates are widely variable between different genetic backgrounds, between chromosomes in the same genome, and even between different regions on the same chromosome [44,45]. ALLMAPS uses a local estimation method based on the positions of the input markers. Cubic spline, which was suggested to be the best method to estimate recombination rate [44], is used to generate interpolation of genetic distance along the chromosome (Figure 8A). Recombination rates are estimated by taking the derivative of the cubic spline (Figure 8B). For each pair of markers that spans a gap, we converted the genetic distance to the physical distance based on the interpolated recombination rate, and then deducted the overhangs (distance from the markers to the edge of the scaffold) to infer the gap sizes (Figure 8C). All pairwise marker combination between two adjacent scaffolds were used in the gap size estimation. The final gap size is inferred based on the smallest estimate among all marker pairs between the flanking scaffolds for all input maps. ALLMAPS offers the gap estimation method as an optional step in the pipeline.

### Data availability

Source code for ALLMAPS are available at [30]. ALLMAPS is available for use through a web-based interface in the iPlant Discovery Environment [46]. Whole genome shotgun raw reads of yellow catfish are deposited under SRA study: SRP050322 ([47]). Input data used in building the yellow catfish and Medicago assembly are available on figshare with the following public DOI:

- Tang, Haibao (2014): ALLMAPS supporting data: Yellow catfish genome assembly [48].
- Tang, Haibao (2014): ALLMAPS supporting data: Medicago genome assembly [49].
